# The Role of Diffusion-Weighted MRI in Correlation with Contrast-Enhanced MRI and Histopathology in the Evaluation of Focal Liver Lesions

**DOI:** 10.7759/cureus.71261

**Published:** 2024-10-11

**Authors:** Mahidhar Varigonda, Jyotsna Yarlagadda, Tarani Chetana Naga Sai, Sujata Patnaik, Sukanya Bhrugumalla, Surya Ramachandra Varma Gunturi

**Affiliations:** 1 Radiology, Nizam's Institute of Medical Sciences, Hyderabad, IND; 2 Gastroenterology, Nizam's Institute of Medical Sciences, Hyderabad, IND; 3 Surgical Gastroenterology, Nizam's Institute of Medical Sciences, Hyderabad, IND

**Keywords:** apparent diffusion coefficient, benign and malignant tumors, contrast enhanced magnetic resonance imaging, diffusion-weighted imaging, focal liver lesions, histopathology, metastasis, primary liver lesions

## Abstract

Introduction: Accurate diagnosis of focal liver lesions is of utmost importance for the initiation of appropriate treatment. This study aims to assess the effectiveness of diffusion-weighted imaging (DWI) in diagnosing focal liver lesions, specifically focusing on differentiating between benign and malignant lesions, distinguishing metastases from primary liver tumors, and identifying various types of both benign and malignant lesions..

Methods: The study design is that of a prospective observational study done on 28 cases with focal liver lesions detected on ultrasound. DWI was done followed by contrast-enhanced MRI (CE-MRI). Identification of the lesion on DWI and apparent diffusion coefficient (ADC) map was made by using the CE-MRI images as a guide. The signal intensity of the lesion on DWI is determined as either diffusion restriction or no restriction. ADC measurements were made quantitatively over various regions of interest (ROIs) of the focal liver lesion. All the lesions were confirmed by histopathological examination (HPE) except few benign lesions like simple cysts and few hemangiomas, which were followed up by ultrasonography after six months.

Results: Our study included 28 patients with 44 focal liver lesions. Out of 28 patients in this study group (n=28), there were a total of 17 male and 11 female patients. The mean age of the sample population was 50.89±13.40 years. The lesions were more commonly seen is the age group of 41-60 years (57.14%). Out of 44 lesions (n), 24 (54.5%) lesions were benign , 20 (45.45%) were malignant. There were 17 hemangiomas (38.6%), 2 hematomas (4.5%), 2 simple cysts (4.5%), 1 hydatid cyst (2.27%), 2 hepatic adenomas (4.5%), 16 hepatocellular carcinoma (HCC) (36.36%), 3 cholangiocarcinoma (6.8%), and 1 hepatoblastoma (2.27%). DWI has 85% sensitivity and 84.7% specificity for the differentiation of benign from malignant lesions. The mean ADC value of benign lesions was 1.83x10^-3 ^mm^2^/s and the mean ADC of malignant liver lesions was 0.96x10^-3 ^mm^2^/s. In the present study, the average cut off of mean ADC to differentiate benign and malignant bone lesions is 1.3x10^-3^ mm^2^/s, with statistically significant p value of 0.001, sensitivity of 95% and specificity of 83.3%.

Conclusion: DWI is a valuable imaging technique in the evaluation of focal liver lesions. The ability to differentiate between benign and malignant liver lesions without the need for contrast is a significant benefit, particularly in challenging cases involving uncooperative patients or when contrast administration is contraindicated.

## Introduction

The liver is the largest and most important internal organ in the human body. It plays a crucial role in supporting immunity against all external threats along with performing functions such as metabolism and digestion. As the liver plays a major role, accurate diagnosis of related abnormalities is important for optimal treatment. Focal liver lesions are one such abnormality commonly observed by radiologists. Focal hepatic lesions, representing a vast spectrum of both benign and malignant lesions that include cysts, abscesses, and hemangioma, pose a challenge in accurate diagnosis and characterization, which is paramount for appropriate treatment. For that accurate diagnosis, numerous imaging techniques have been invented and, among them, diffusion-weighted MRI (DW-MRI) has recently emerged as a promising method. DW-MRI utilizes the intrinsic molecular motion of water molecules within tissues to provide functional and structural information. DW-MRI is different from contrast-enhanced MRI (CE-MRI), which relies on intravenous contrast agents. Use of DW-MRI represents an advancement in technology in liver imaging, offering enhanced diagnostic accuracy, decreased patient risk, and an increase of applicability across diverse patient populations.

In assessing and diagnosing focal hepatic lesions, DW-MRI has numerous advantages compared to traditional CE-MRI. By utilizing the diffusion properties of water molecules, DW-MRI provides helpful and informative insights into tissue microstructure and cellularity. Based on the ability to distinguish differences in cell densities and tissue architecture, DW-MRI is capable of differentiating benign from malignant lesions. Studies have demonstrated that DW-MRI can achieve comparable or even superior diagnostic accuracy compared to CE-MRI, especially in lesions where contrast uptake may be heterogeneous or equivocal [1].

By analyzing the random motion of water molecules within tissues using diffusion-sensitizing gradients, DW-MRI creates diffusion-weighted images, which can be used to provide quantitative metrics such as apparent diffusion coefficient (ADC) maps. These maps represent the tissue cellularity and integrity, which can be used to outline the hepatic lesions. Recent advancements in MRI technology, particularly in enhancing field strengths and gradient systems, have helped obtain improved spatial and temporal resolution of the diffusion-weighted images produced via DW-MRI, which significantly enhances diagnostic performance [2].

Clinical studies have substantiated the utility of DW-MRI across a spectrum of hepatic lesions. For example, DW-MRI has shown great strides in identifying tiny lesions and differentiating them from benign regenerating nodules in the evaluation of hepatocellular carcinoma (HCC). Furthermore, in the setting of metastatic liver disease, the capacity of DW-MRI to identify minute alterations in lesion cellularity has proven outstandingly helpful for response evaluation and treatment planning. Additionally, by adding functional information to traditional morphological imaging, DW-MRI has shown potential in reducing needless biopsies [3].

While there are advantages to using DW-MRI, there are also limitations. Particularly, technical limitations such as susceptibility artifacts, motion artifacts, and variability in ADC measurements across different MRI systems can greatly affect image quality and diagnostic reliability, thereby decreasing accuracy. Additionally, a significant learning curve is required to interpret DW-MRI findings, requiring expertise in advanced imaging techniques, which may limit its widespread adoption in clinical practice in the short run. While DW-MRI is highly advantageous for patients with renal impairment or contrast allergies, for who cannot undergo CE-MRI, its application is still a topic of research in specific clinical scenarios, such as assessing lesion vascularity, which remains a subject of ongoing debate [4].

Ongoing research shows the potential for further enhancing the diagnostic capabilities of DW-MRI in hepatic imaging. Every day, there are advancements in technologies, which may also aid the research in improving and refining quantitative diffusion metrics while simultaneously exploring novel MRI sequences and integrating artificial intelligence algorithms that can help in automated lesion characterization. The integration of DW-MRI into regular clinical practice will slowly transform the diagnosis and treatment of localized hepatic lesions parallel to this field’s evolution, increasing the precision of individualized treatment plans catered to the specific requirements of each patient [5].

The aims and objectives of the study were to evaluate the role of diffusion-weighted imaging (DWI) in the diagnosis of focal liver lesions, to differentiate benign from malignant liver lesions by using DWI and subsequently correlate with CE-MRI, to differentiate liver metastasis from primary liver lesions by using DWI and subsequently correlate with CE-MRI and to differentiate among various benign and malignant lesions.

## Materials and methods

The study was conducted at the Department of Radiology and Imageology in Nizam’s Institute of Medical Sciences, Hyderabad, and was approved by the Institutional Ethics Committee. This was a prospective observational study, conducted over a period of 19 months (March 2020-October 2021). 31 patients with focal liver lesions detected on ultrasound abdomen, irrespective of age, were referred from the gastroenterology departments to ours, out of which three were excluded from the study as they did not undergo DWI. A total of 28 patients were included in the present study.

The study was performed using 3T MRI Siemens Magnetom Skyra (48-channel machine) with 18 channel body coils.

Before DWI, several MRI sequences were conducted. One was coronal T2-weighted half-Fourier single-shot turbo spin-echo (HASTE), with the following parameters: repetition time (TR) of 2,000 ms, echo time (TE) of 91 ms, flip angle (FA) of 160°, slice thickness of 5 mm, distance factor of 10%, and field of view (FOV) of 350 mm. Another was gradient-echo T1 sequences for both in-phase and out-of-phase imaging, using a TR of 4.34 ms, in-phase TE of 2.76 ms, out-of-phase TE of 1.34 ms, FA of 9.0°, slice thickness of 5 mm, interslice gap of 20%, and FOV of 380 mm. Yet another was axial respiratory-triggered turbo spin-echo T2-weighted sequence with fat saturation, employing a TR of 2,000 ms, TE of 91 ms, slice thickness of 5 mm, interslice gap of 20%, and FOV of 380 mm.

DW-MRI was performed before dynamic imaging, using a single-shot spin-echo echo planar imaging sequence with the b factors of 50, 400, and 800 mm^2^/s along the three orthogonal directions. The sequence was obtained using the following technical parameters: TR = 6,600 ms, TE = 48 ms, slice thickness = 5 mm with inter-slice gap = 20%, FOV = 380 mm, and fat saturated. The ADC map images were created automatically by the system.

Then dynamic contrast-enhanced axial T1-weighted three-dimensional volumetric interpolated breath hold examination sequences were obtained with the following parameters: TR = 3.31 ms, TE = 1.3 ms, FA = -9°, and slice thickness = 3 mm. The study was conducted following a bolus injection of 0.1 mmol/kg of gadobenate dimeglumine (MULTIHANCE®) administered at a rate of 2 ml/s, which was then flushed with 20 ml of sterile 0.9% saline solution into the antecubital vein. The injection of contrast media and saline solution was performed manually. Dynamic imaging using the T1 technique was performed via the triphasic method (arterial phase (20-30 s), porto-venous phase (60-70 s), and delayed equilibrium phase (3-5 min)) after administration of contrast media. The obtained results were correlated with HPE confirmation in suspected neoplastic lesions.

Lesion characterization was initially conducted on conventional MRI. The lesion was determined on the DWI and ADC map by using the conventional MRI as a guide. On the DWIs (b 50, 400, and 800), signal intensity of lesions was subjectively determined as either hypointense (free diffusion) or hyperintense (restricted diffusion) via visual comparison with the adjacent (normal) liver parenchyma. Quantitative measurements of the ADC were made on the ADC map using electronic cursors in different regions of interest (ROIs) on the focal liver lesion, specifically in the solid areas of the lesion and preferably where diffusion restriction was shown, while avoiding cystic/necrotic areas to prevent underestimation of the cellularity of the tumor. Only lesions more than 1 cm were considered for ADC evaluation, as the ROI for each lesion needed to be placed at least three times. All ROIs were almost identical in size and shape. The mean ADC value of the lesion was calculated from the maximum and minimum ADC values in different regions of the lesion, as derived from ROIs. The ADC values were expressed in x10^-3^ mm^2^/s. All the cases underwent fine needle aspiration cytology (FNAC)/biopsy and were confirmed via HPE (the gold standard for confirmation of diagnosis) except for a few benign stable lesions, which underwent follow-up scan with ultrasonography after six months, demonstrating typical appearance (like simple cysts and classical hemangiomas).

Statistical analysis was performed using the Statistical Package for Social Sciences (SPSS) software, version 21. ADC values between the two groups (benign and malignant bone tumors and infection and malignant bone tumors) were compared using Student’s t test. Receiver operating characteristic curves were performed and area under curve was calculated to seek the best cutoff values of mean ADC. Sensitivity and specificity were calculated when employing the cut-off mean ADC values. Area under curve of >0.5 was considered statistically significant.

## Results

**Figure 1:**
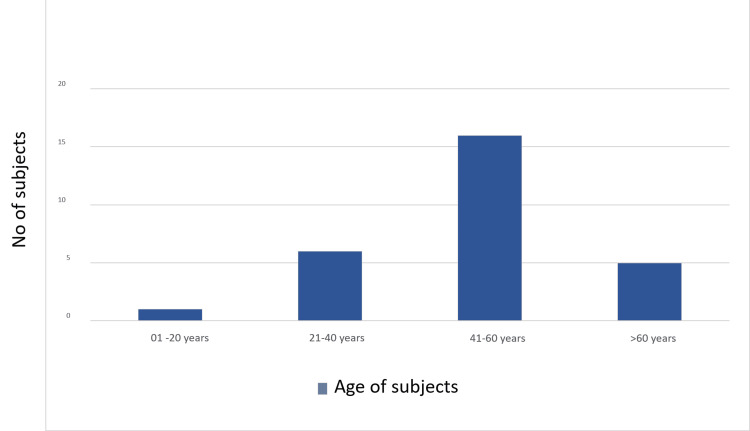
Bar graph showing age distribution

The study included 28 patients with clinical suspicion of and/or detection of focal liver lesions via ultrasonography, irrespective of the age group and gender. The study group consisted of 17 males and 11 females, with a mean age of 50.89 years (range: 19-71 years). The most common age group was 41 to 60 years, followed by 21 to 40 years (Figure 1). The mean and median age of patients with benign focal liver lesions were 49.07 and 50 years, respectively. The mean and median ages of patients with malignant focal liver lesions were 52.7 and 57.5years, respectively. The lowest age observed in the study was 19 years (hepatoblastoma). The highest age was 71 years (hemangioma), followed by 70 years (HCC).

**Figure 2:**
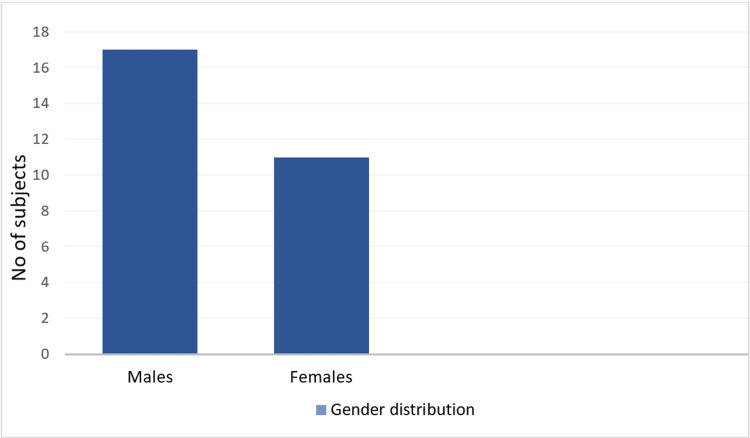
Bar graph showing gender distribution

 We observed a greater number of males (n = 17; 60.71%) than females (n = 11; 39.28%), with a male to female ratio of 1.5:1 (Figure 2).

**Figure 3:**
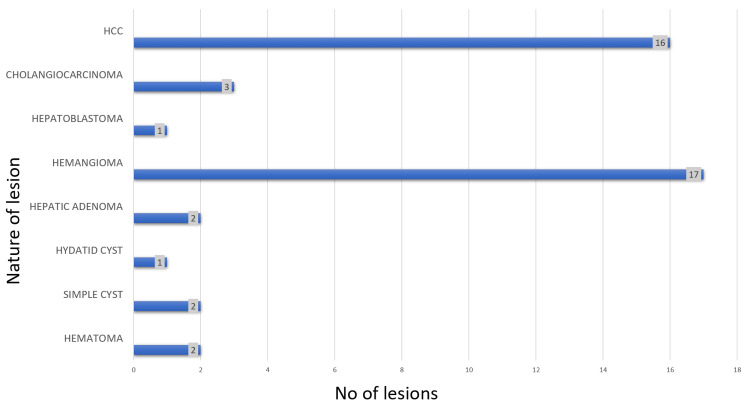
Bar diagram showing number of lesions in each spectrum HCC: Hepatocellular carcinoma

**Figure 4:**
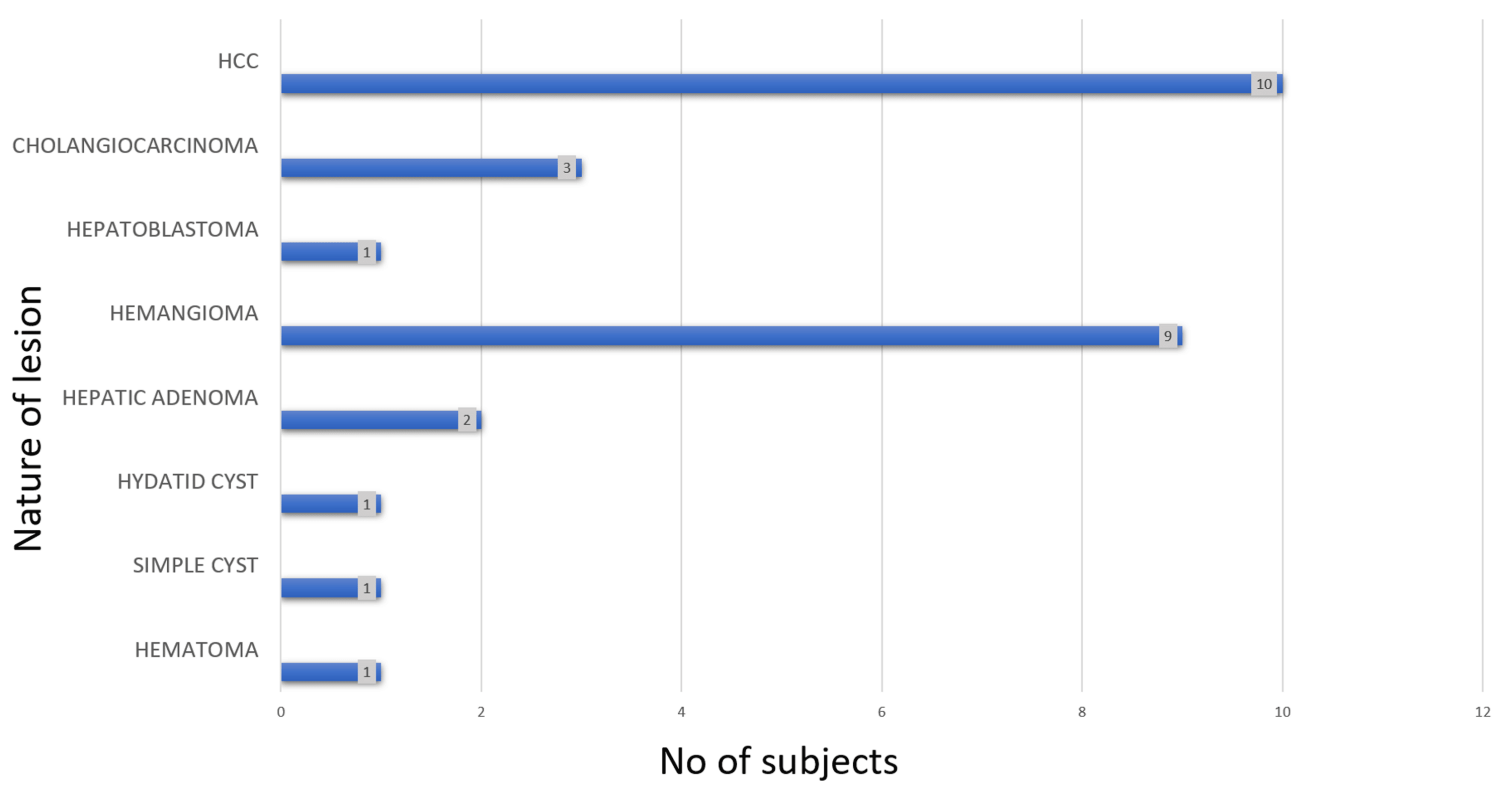
Bar diagram showing spectrum of lesions in patients HCC: Hepatocellular carcinoma

A total of 28 patients and 44 lesions were included in this study (Figure 3). Among the 28 patients, 14 (50%) were found to have benign lesions and 14 (50%) had malignant lesions. The most common benign lesion was hemangioma, found in 9 patients, and the most common malignant lesion was HCC, found in 10 patients (Figure 4). 21 (75%) of the 28 patients had solitary lesions, and 7 (25%) patients had multiple (Table 1). Multiplicity was common in patients with hemangiomas (five patients). The most common lesions in males were malignant (70.5%) and the most common lesions in females were benign (47.05%). HCC was relatively more common in males and hepatic adenoma was relatively more common in females (Table 2). The largest lesion was an HCC, measuring 15.6 cm, while the smallest lesion was a hemangioma, measuring 1.1cm. The average size across all lesions was 5.1 cm.

**Table 1:** Multiplicity of lesions HCC: Hepatocellular carcinoma

Type of lesion	Number of patients	Number of lesions
HCC	2	5
Hemangiomas	3	8
Hemangioma+ hepatoma	1	5 (4 hemangiomas + 1 hepatoma)
Hemangioma + simple cyst	1	2 (1 hemangioma + 1 simple cyst)

**Table 2:** Gender distribution of lesions HCC: Hepatocellular carcinoma

Spectrum of lesions	Males	Females
HCC	9	1
Cholangiocarcinoma	3	0
Hepatoblastoma	0	1
Hemangioma	4	5
Hepatic adenoma	0	2
Hydatid cyst	0	1
Simple cyst	0	1
Hepatoma	1	0

Qualitative analysis: diffusion restriction in focal liver lesions

As described, diffusion restriction was visually analyzed across all three b values (50, 400, and 800 mm^2^/s). However, results with a b value of 800 mm^2^/s were more specific. Qualitative analysis of signal intensities on DWI were divided into restricted, partly restricted, and not restricted (Table 3).

**Table 3:** Signal intensities of various lesions on DWI and ADC maps HCC: Hepatocellular carcinoma; DWI: Diffusion-weighted imaging; ADC: Apparent diffusion coefficient

Type of lesion	Restricted signal intensity	No restriction	Partly restricted
HCC	11	0	5
Cholangiocarcinoma	3	0	0
Hepatoblastoma	0	0	1
Hemangioma	1	12	4
Hepatic adenoma	0	0	2
Hydatid cyst	0	1	0
Simple cyst	0	2	0
Hematoma	0	1	1
TOTAL	15	16	13

**Figure 5:**
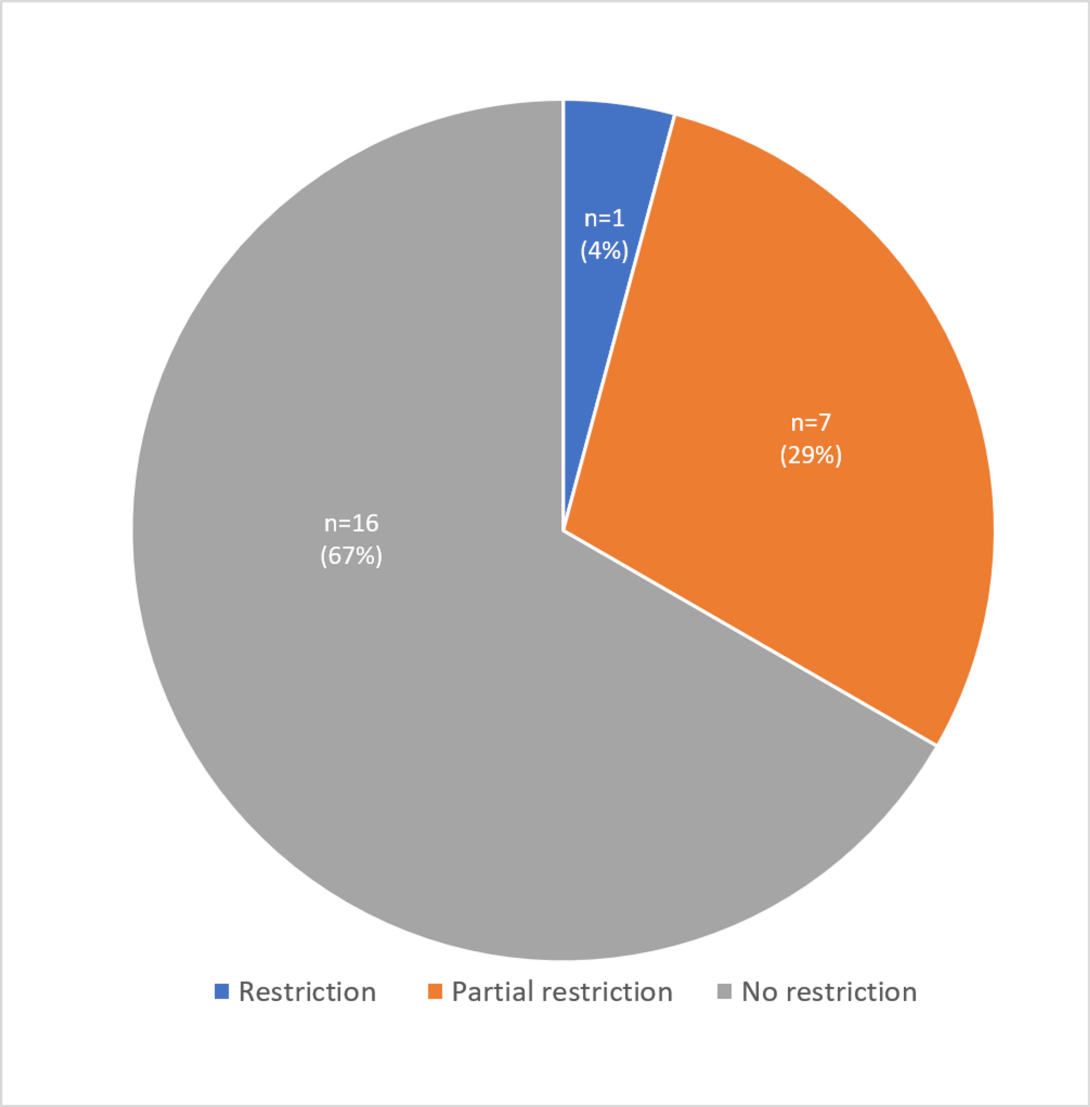
Pie diagram showing pattern of diffusion restriction in benign lesions

Of the 44 lesions included in this study, 15 showed diffusion restriction (hyperintense on DWI with reversal on ADC), 13 were partially restricted (patchy areas of hyperintensities on DWI with corresponding areas showing ADC reversal), and 16 lesions showed no diffusion restriction. 11 out of 16 (68.75%) HCC lesions showed diffusion restriction, with 5 (31.25%) demonstrating partial restriction. Hepatoblastoma and all three of the cholangiocarcinomas were restricted (100%), while four (23.5%) hemangiomas were partly restricted and one (5.8%) showed diffusion restriction. Most (12, 70.5%), showed no restriction on DWI with hyperintense signal on DWI and ADC maps, which might be due to T2 shine through effect. The two (100%) hepatic adenomas showed partial restriction on DWI. The hydatid cyst and simple cysts showed no diffusion restriction (100%) and, out of the two hematomas, one (50%) showed T2 shine through effect and the other (50%) showed partial diffusion restriction. Of the total 24 benign liver lesions 67% lesions showed no restriction on DWI, 29% lesions showed partial restriction and 4% lesions showed diffusion restriction on DWI (Figure 5).

**Figure 6:**
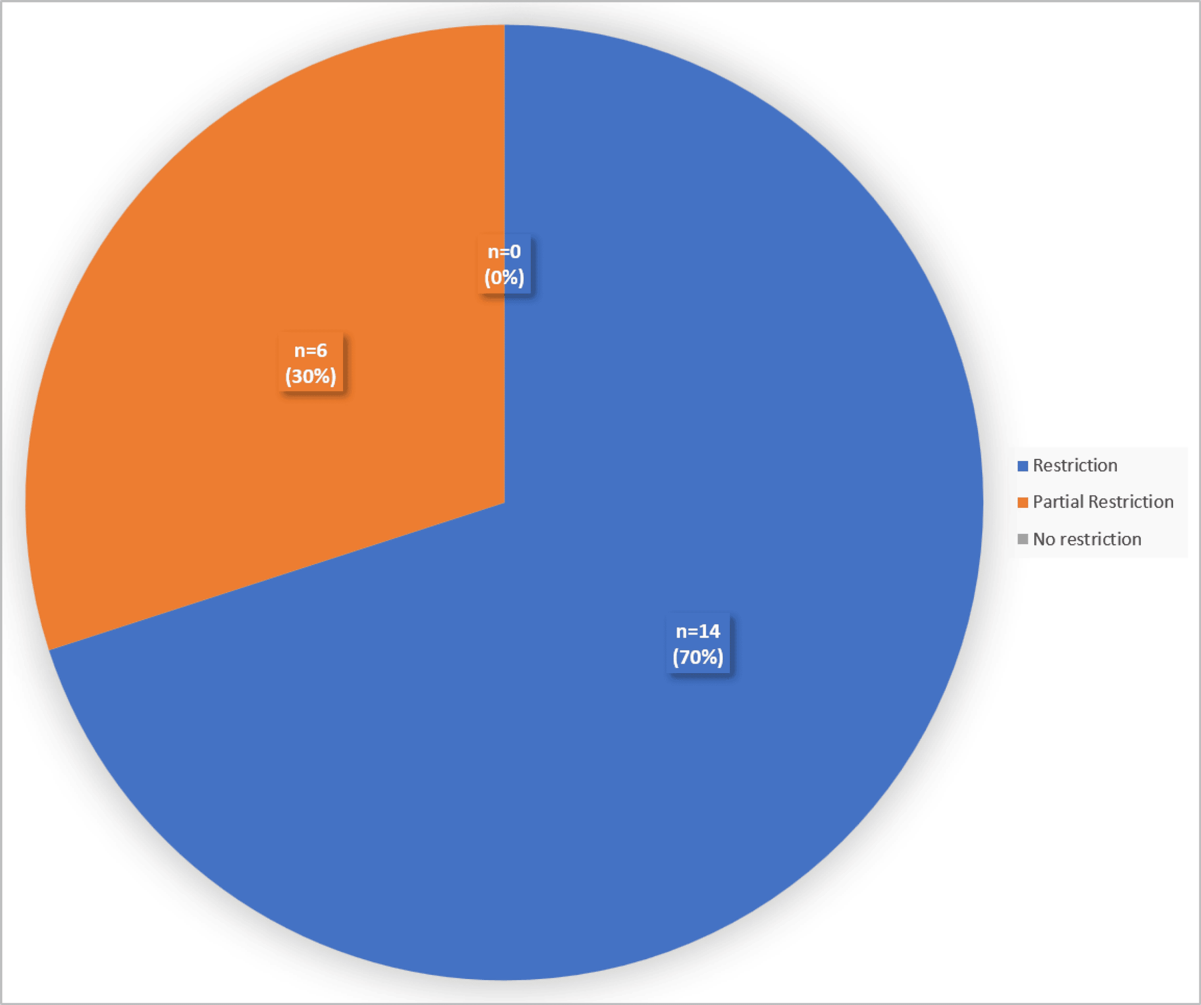
Pie diagram showing diffusion restriction pattern in malignant lesions

From a total of 20 malignant liver lesions, 70% showed restriction on DWI and 30% showed partial restriction (Figure 6).This study has shown that DWI has an accuracy rate of 79.4% in detecting benign lesions and 84% in detecting malignant liver lesions.

Quantitative analysis of DWI with mean ADC values

The highest mean ADC value noted among the benign liver lesions was that of the simple hepatic cyst (3.2 x 10^-3^ mm^2^/s), followed by the hydatid cyst (2.6 x 10^-3^ mm^2^/s). The lowest mean ADC value noted in the benign liver lesions was hemangioma (0.79 x 10^-3^ mm^2^/s). HCC had the highest (1.4 x 10^-3^ mm^2^/s) and the lowest (0.68 x 10^-3^ mm^2^/s) ADC values among malignant lesions. The mean ADC value of benign lesions was 1.83 ± 0.6 x 10^-3^ mm^2^/s, while the mean ADC value of malignant lesions was 0.96 ± 0.16 x 10^-3^ mm^2^/s (Table 4). According to Table 4 ,the lowest mean ADC value among the different spectrum of malignant lesions was shown by cholangiocarcinoma (0.92 x 10^-3 ^mm^2^/s) and the highest was exhibited by hepatoblastoma (1.18 x 10^-3^ mm^2^/s). Among the benign lesions, the lowest mean ADC value was seen in hepatic adenomas (1.1 x 10^-3 ^mm^2^/s) and the highest was in simple hepatic cysts (3.1 x 10^-3^ mm^2^/s). The minimum ADC value of different types of liver lesions was seen in HCC (0.10 x 10^-3^ mm^2^/s), followed by cholangiocarcinoma (0.12 x 10^-3^ mm^2^/s). The maximum ADC value was seen in hydatid cysts (3.73 x 10^-3^ mm^2^/s), followed by the simple cyst (3.43 x 10^-3^ mm^2^/s). The average cutoff of mean ADC, to differentiate benign and malignant bone lesions, was 1.30 x 10^-3 ^mm^2^/s, with a statistically significant p value of 0.001, sensitivity of 95%, and specificity of 83.3%. Although there was some degree of overlap between benign and malignant liver lesions, most of the lesions could be differentiated based on mean ADC values. As the p value was <0.05, that is, 0.0001, the mean ADC cutoff was considered statistically significant. The accuracy of DWI in differentiating benign from malignant lesions at this cutoff was 88.63%.

**Table 4:** Different spectrum of lesions and their mean ADC values HCC: Hepatocellular carcinoma; ADC: Apparent diffusion coefficient

Type of lesion	Number of lesions	Mean ADC value (mm^2^/s)	Standard deviation	Standard error	Confidence interval (95%)		Minimum ADC value	Maximum ADC value
Cholangiocarcinoma	3	0.92	0.12	0.0702	0.621	1.218	0.12	1.97
HCC	16	0.96	0.20	0.0502	0.853	1.066	0.10	2.12
Hepatoblastoma	1	1.1	-	-	-	-	0.42	1.72
Hemangioma	17	1.74	0.42	0.1036	1.524	1.955	0.51	3.01
Hepatic adenoma	2	1.18	0.11	-	-	-	0.83	1.43
Hematoma	2	1.58	0.52	-	-	-	0.31	2.66
Simple cyst	2	3.11	0.14	-	-	-	2.94	3.43
Hydatid cyst	1	2.6	-	-	-	-	1.54	3.73

Benign liver lesions

Simple Cyst

**Figure 7:**
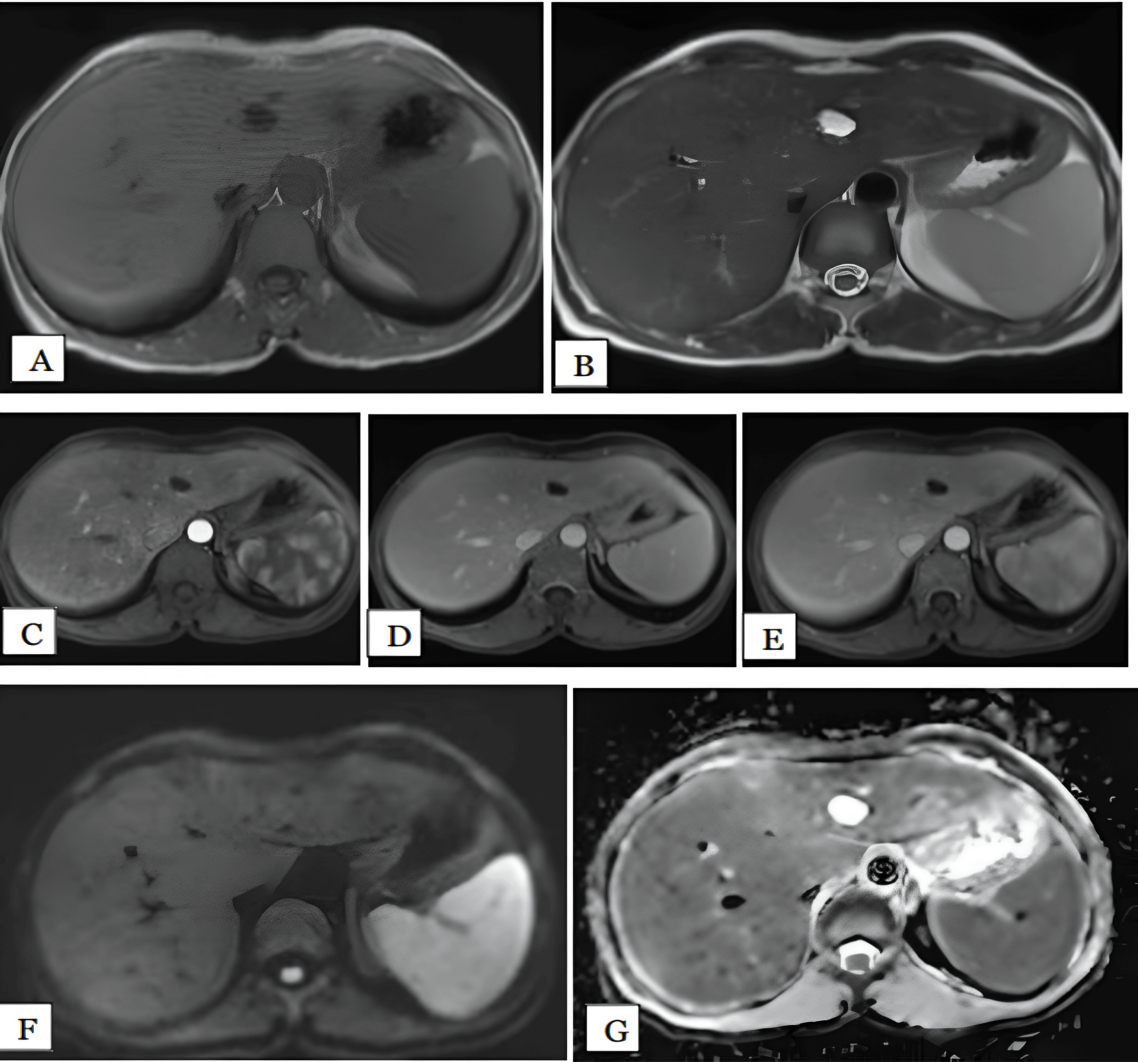
Simple hepatic cyst in a 44-year-old female Axial T1 (A), T2 (B), arterial (C), portal, (D), and delayed (E) phase MRI images show well-defined T1 homogenous hypointense. T2 homogenous hyperintense lesion was noted in segment IVa of left lobe of liver showing no enhancement post contrast. DWI (F) and ADC (G) maps show the lesion is isointense on DWI with hyperintense on ADC maps with mean ADC value of 3.2 x 10^-3 ^mm^2^/s, suggestive of facilitated diffusion. DWI: Diffusion-weighted imaging; ADC: Apparent diffusion coefficient

We had two patients with simple cysts: one involving segment IV of the left lobe of the liver and the other involving segment VIII of the right lobe, measuring 1.4 and 2.3 cm, respectively. Both lesions (100%) were hypointense on T1 and hyperintense on T2-weighted images with no post contrast enhancement (Figure 7). Ultrasonogram showed anechoic simple cystic lesions. MRI had 100% sensitivity in diagnosing simple hepatic cysts. No restriction was noted in DWI, therefore, DWI had 100% sensitivity in diagnosing simple hepatic cysts. The mean ADC values of the lesions was 3.12 x 10^-3^ mm^2^/s.

Hydatid Cyst

**Figure 8:**
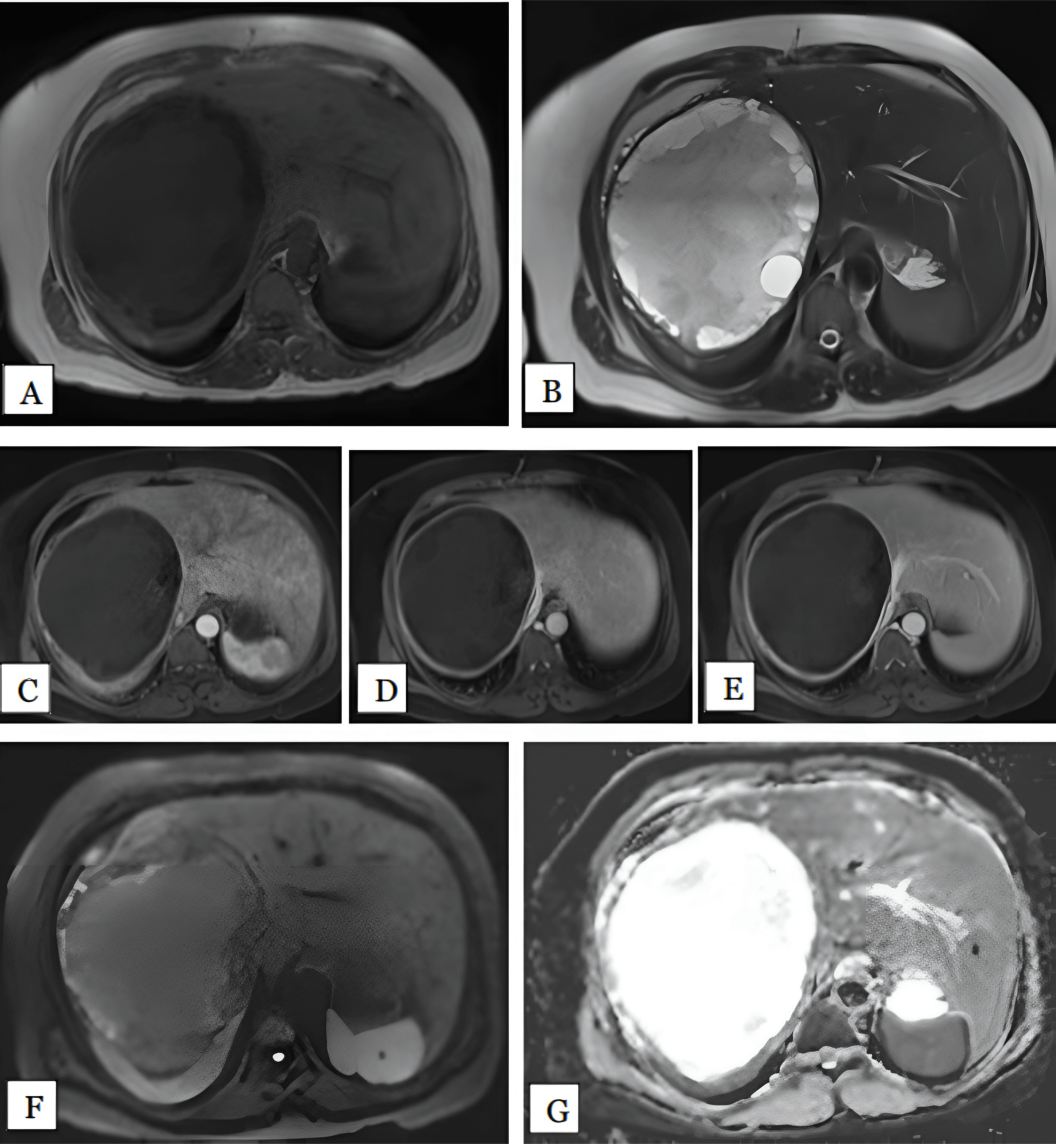
Hydatid cyst in a 50-year-old female Axial T1 (A), T2 (B), arterial (C), portal (D), and delayed (E) phase MRI images show well-defined large T1 hypointense. T2 hyperintense lesion with peripherally arranged small hyperintense cystic areas were noted in segment VII, VIII of right lobe of liver, showing peripheral rim enhancement post contrast. HPE confirmed the diagnosis of hydatid cyst. DWI (F) and ADC (G) maps show the lesion is isointense on DWI with peripheral hyperintensity and hyperintense on ADC maps with mean ADC value of 2.6 x 10^-3^ mm^2^/s, suggestive of facilitated diffusion. HPE: Histopathological examination; DWI: Diffusion-weighted imaging; ADC: Apparent diffusion coefficient

We had only one lesion of hydatid cyst, in a 50-year-old female patient. The lesion was noted in the right lobe involving the V, VII, and VIII segments. The lesion measured 11 cm. It was hypointense on T1 and hyperintense on T2-weighted images with multiple peripherally arranged small daughter cysts (Figure 8). The lesion showed peripheral rim enhancement post contrast. No diffusion restriction was noted on DWI. The mean ADC value of lesion was 2.62 x 10^-3^ mm^2^/s.

Hematoma

**Figure 9:**
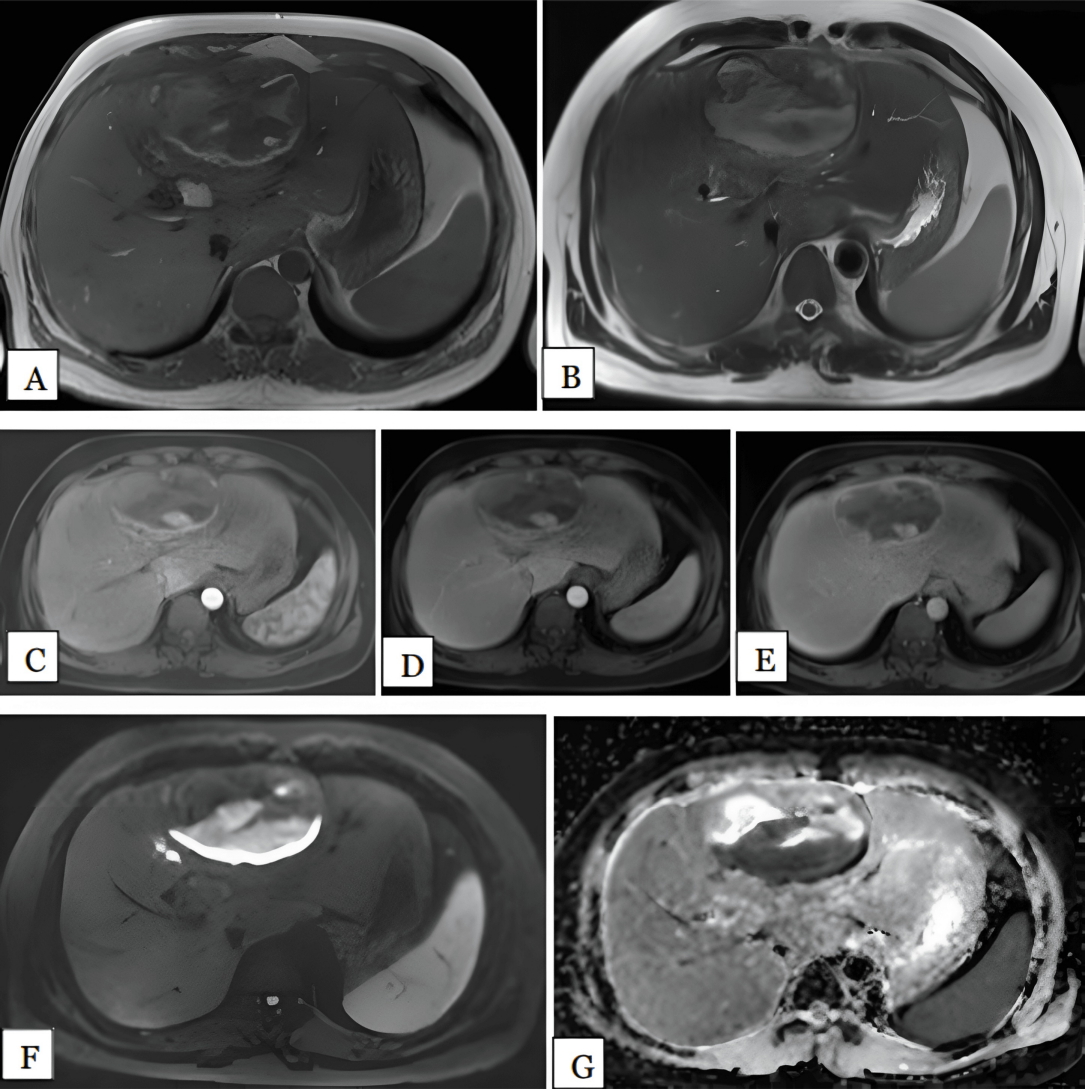
Hematoma in a 36-year-old male Axial T1 (A), T2 (B), arterial (C), portal (D), and delayed (E) phase MRI images show well-defined lobulated lesion. Heterogenously hyperintense on both T1 and T2 measuring 10 x 8 cm were noted involving the segment IVa and IIb. Post contrast showed no enhancement. DWI (F) and ADC (G) maps showed the part of the lesion is hyperintense on DWI with reversal on ADC maps with mean ADC value of 1.1 x 10^-3 ^mm^2^/s, suggestive of partial restriction. DWI: Diffusion-weighted imaging; ADC: Apparent diffusion coefficient

We had two patients of liver hematomas: one male (50%) (Figure 9) and one female (50%), with both involving the left lobe of the liver, one on segment IV and the other on segment IV and II. The largest lesion measured 10 cm. One patient had a history of right hepatectomy for multiple hemangiomas. One lesion was heterogeneously hyperintense on both T1- and T2-weighted images with no enhancement post contrast. The other lesion was hypointense on T1 with a peripheral thick hyperintense rim and hyperintense on T2 with a peripheral hypointense rim. In this case, post contrast showed no enhancement. Therefore, MRI had 100% sensitivity in diagnosing hematomas. One lesion showed partial restriction on DWI and the other showed no restriction. Therefore, DWI had 75% sensitivity. The mean ADC value was 1.58 x 10^-3 ^mm^2^/s.

Hemangiomas

**Figure 10:**
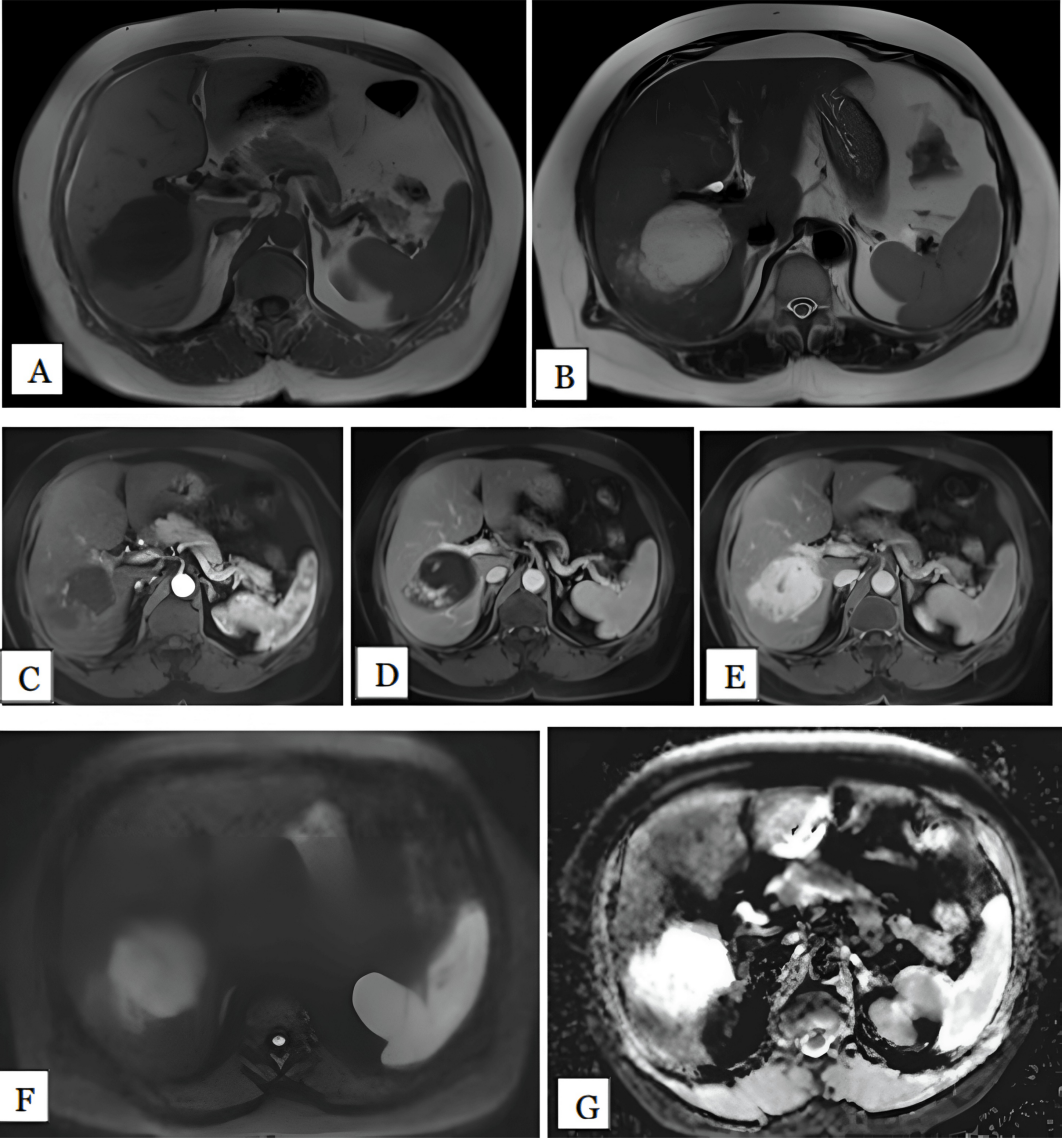
Hemangioma in a 60-year-old female Axial T1 (A), T2 (B), arterial (C), portal (D), and delayed (E) phase MRI images show well-defined lobulated lesion, which is T1 hypointense and T2 hyperintense in right lobe of liver segment VI & VII abutting right portal vein. In arterial phase, the lesion showed peripheral nodular enhancement with progressive centripetal filling of the lesion in portal phase with heterogenous enhancement of entire lesion in delayed phase. HPE confirmed the diagnosis of hemangioma. DWI (F) and ADC (G) maps show the lesion is hyperintense on DWI with hyperintense on ADC maps with mean ADC value of 2.1 x 10^-3 ^mm^2^/s, suggestive of T2 shine through (no restriction). HPE: Histopathological examination; DWI- Diffusion weighted imaging; ADC: Apparent diffusion coefficient

We had 9 patients with hemangiomas and a total of 17 lesions. Of these patients, five (55.5%) were female and four (44.4%) were male. Three (33.3%) patients had multiple lesions, one (11.1%) had two lesions, and the remaining five(55.5%) patients had solitary lesions. Five patients (55.5%) had right lobe involvement, three (33.3%) had the involvement of both lobes, and one (11.1%) had left lobe involvement. The largest hemangioma was 14 cm and the smallest was 1.1 cm. Among all 17 lesions studied in these 9 patients, 4 (23.5%) were giant hemangiomas (more than 6 cm), with the largest measuring 14 cm and the smallest 7.1 cm. All the lesions were hypointense on T1-weighted images and hyperintense on T2-weighted images (Figure 10).

16 of these 17 lesions (94.1%) showed typical peripheral nodular enhancement in the arterial phase with progressive centripetal filling in delayed images, while 1 lesion (5.9%) showed patchy heterogenous enhancement in the arterial phase with complete filling of the lesion in the delayed phase. All the lesions were accurately characterized on MRI as hemangiomas, based on the imaging characteristics. Therefore, MRI had 100% sensitivity in diagnosing hemangiomas. 12 out of 17 (70.5%) lesions showed no restriction on DWI with hyperintense signal on DWI and ADC maps, which might be due to T2 shine through effect. Four (23.5%) lesions were partially restricted and one (5.8%) showed diffusion restriction. Hence, DWI had a sensitivity of 82.3% in diagnosing hemangiomas. The mean ADC value of hemangiomas was 1.74 x 10^-3^ mm^2^/s.

Hepatic Adenoma

**Figure 11:**
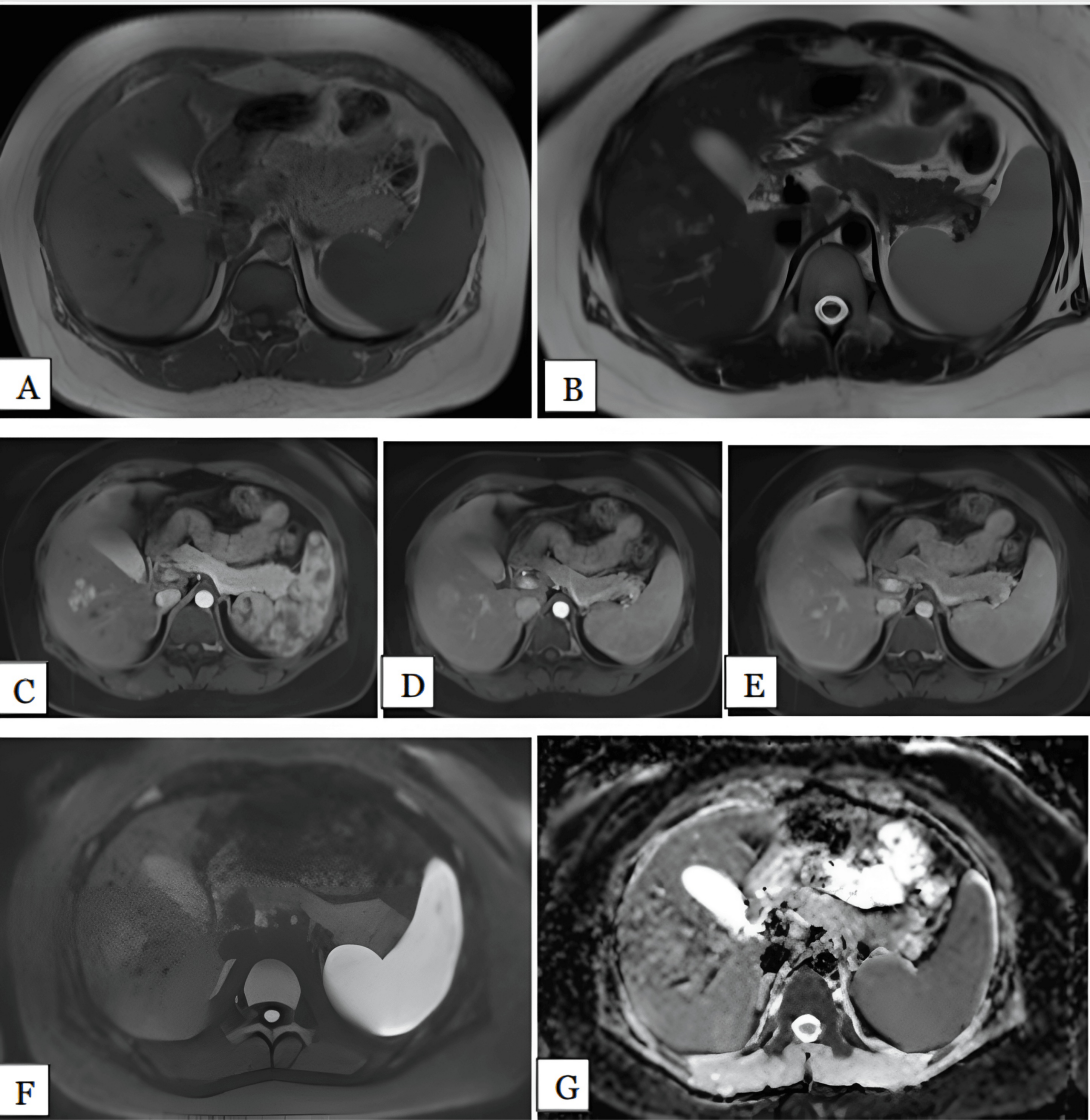
Hepatic adenoma in a 24-year-old female Axial T1 (A), T2 (B), arterial (C), portal (D), and delayed(E) phase MRI images show T1 isointense, T2 slightly hyperintense lesion with noted in segment VII of liver. Lesion was heterogeneous hyperintense in arterial phase, progressive washout in portal venous, delayed phases and becomes isointense to liver in delayed phase. HPE confirmed the diagnosis of hepatic adenoma. DWI (F) and ADC (G) maps showing the lesion is mild hyperintense on DWI with isointense on ADC maps with mean ADC value of 1.1 x 10^-3 ^mm^2^/s, suggestive of partial restriction. HPE: Histopathological examination; DWI: Diffusion-weighted imaging; ADC: Apparent diffusion coefficient

We had two patients with hepatic adenomas (Figure 11). Both were females in the age group of 21 to 40 years, whereby one had a history of contraceptive pill intake. One lesion, the largest in this pair, was 3.3 cm, while the other was 2.7 cm. Both involved segment VII of right lobe of the liver. In one patient, the lesion was isointense on T1 in and out of phase and slightly hyperintense on T2 and showed heterogenous enhancement in the arterial phase with mild washout in the porto-venous phases, becoming isointense to liver in the delayed phase. In another patient, the lesion was iso to hypointense on T1 in phase and hyperintense on T1 out of phase, with suppression of background fatty liver, and hypointense on T2 with slight hyperintensity on T2 fat saturated sequence. Post contrast, this lesion showed homogenous enhancement in the arterial phase with washout in the portal phase, and subsequently became isointense in the delayed phase. The two lesions were characterized as adenomas on MRI. On FNAC, the two lesions were proved to be hepatic adenomas. MRI had 100% sensitivity in diagnosing adenomas, while the two lesions showed partial restriction on DWI. Hence, DWI had lower sensitivity in diagnosing adenomas. The mean ADC value of the adenomas was 1.18 x 10^-3^ mm^2^/s.

Malignant liver lesions

HCC

This study included 10 patients with HCC, 9 (90%) were in the age group of 50 to 70 years and only 1 was in the age group of under 30 years. A total of 16 lesions were included, whereby 2 patients had multi-centric HCC lesions. The largest lesion measured 15.6 cm and the smallest was 1.3 cm. In terms of location, 12 (75%) lesions were in the right lobe, 2 (12.5%) were in the left lobe, and 2 (12.5%) lesions involved both lobes. 

**Figure 12:**
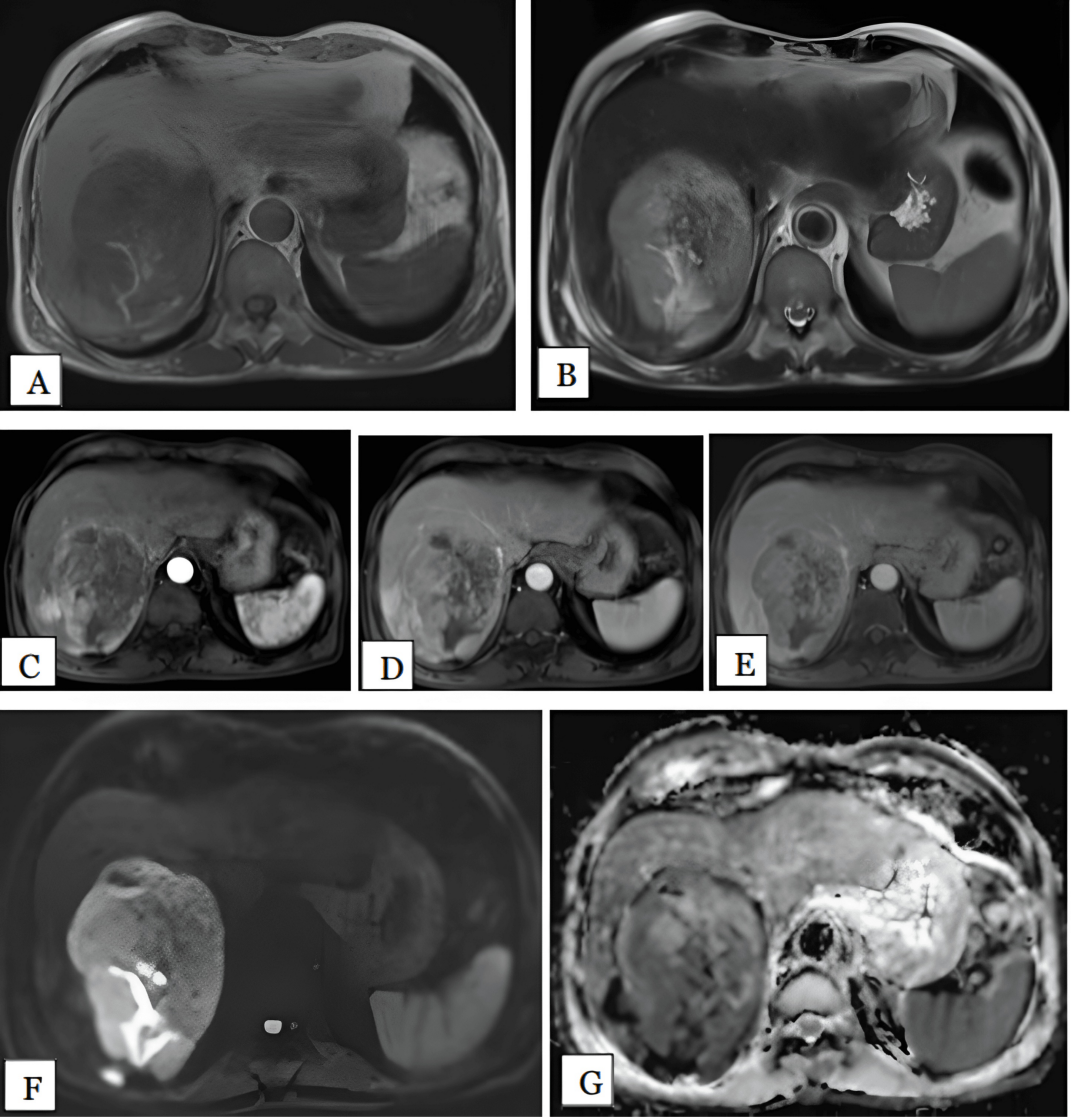
HCC in a 57-year-old male Axial T1 (A), T2 (B), arterial (C), portal (D), and delayed(E) phase MRI images show well-circumscribed lobulated iso-hypointense lesion on T1 with areas of hyperintensities (hemorrhage). Heterogeneously hyperintense on T2 was noted in segments V, VI, VII of right lobe of liver. The lesion is showed heterogeneous enhancement on arterial phase with few non-enhancing areas within and there is progressive wash out on portal phase with central washout with persistent peripheral capsular rim enhancement. HPE confirmed the diagnosis of HCC. DWI (F) and ADC (G) maps show the lesion is hyperintense on DWI with patchy areas of reversal on ADC maps with mean ADC value of 1.2 x 10^-3 ^mm^2^/s, suggestive of restriction. HPE: Histopathological examination; HCC: Hepatocellular carcinoma; DWI: Diffusion-weighted imaging; ADC: Apparent diffusion coefficient

On T1-weighted images, 13 lesions (81.25%) were hypointense, 2 (12.5%) were isointense, and 1 lesion (6.25%) was slightly hyperintense . On T2, 15 (93.75%) lesions were hyperintense and 1 (6.25%) lesion was isointense. 14 lesions (87.5%) showed heterogenous enhancement in arterial and portal phases with washout in delayed phase, and 2 lesions showed persistent enhancement in delayed phases (12.5%), which were characterized on MRI as metastasis with a history of adenocarcinoma stomach. On MRI, 14 out of 16 lesions were correctly diagnosed as HCC, with 1 cholangiocarcinoma falsely diagnosed as HCC, therefore, MRI had a sensitivity of 87.5%. On DWI, 11 lesions showed restriction, with 5 lesions showing partial restriction, therefore, DWI had an 84.3% sensitivity in diagnosing HCC in our study. The mean ADC value was 0.96 x 10^-3^ mm^2^/s. (Figure 12)

Concerning demonstrating pseudo capsule, 9 (56.25%) out of 16 lesions did so. All the pseudo capsules were isointense to hypointense on T1-weighted images and slightly hyperintense on T2-weighted images, showing enhancement in delayed phase images.

Cholangiocarcinoma

**Figure 13:**
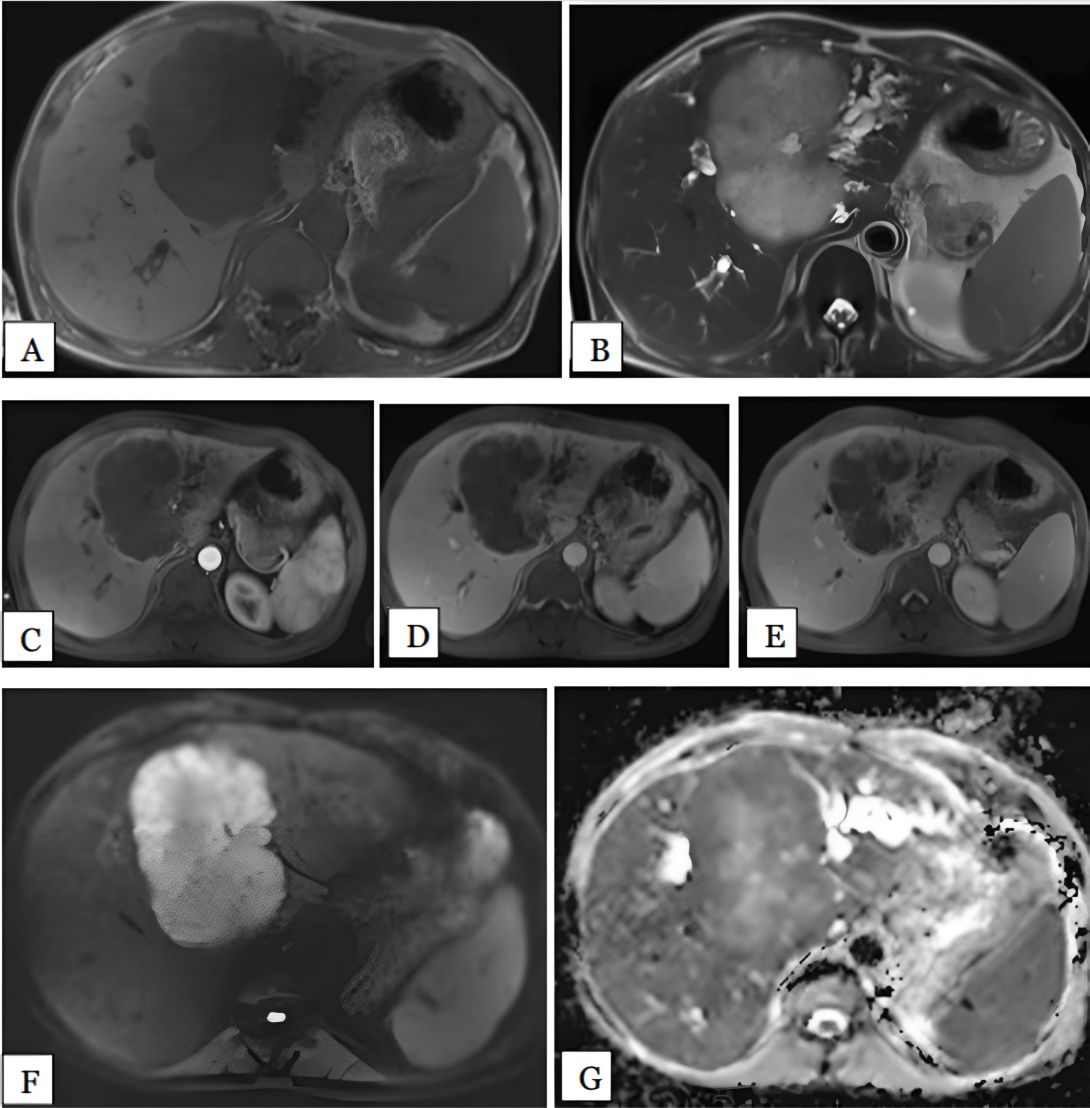
Cholangiocarcinoma in a 48-year-old male Axial T1 (A), T2 (B), arterial (C), portal (D) and delayed (E) phase MRI images show well-circumscribed lobulated T1 hypointense. T2 heterogenous hyperintense lesion was noted in segment IV of left lobe of liver compressing CBD at hilum causing right and left IHBRD. The lesion shows peripheral rim enhancement in arterial phase with progressive peripheral heterogenous enhancement in portal and delayed phases. HPE confirmed the diagnosis of cholangiocarcinoma. DWI (F) and ADC (G) maps show the lesion is hyperintense on DWI predominantly periphery with reversal on ADC maps with mean ADC value of 0.98 x 10^-3 ^mm^2^/s giving target sign, suggestive of restriction. HPE: Histopathological examination; DWI: Diffusion-weighted imaging; ADC: Apparent diffusion coefficient; CBD: Common bile duct

We had three patients of intrahepatic cholangiocarcinomas, of which one was noted at hepatic hilum and another involved segment V and VI on the right lobe of liver. The third lesion involved segment IV on the left lobe of liver. All three lesions (100%) were hypointense on T1 and heterogenous hyperintense on T2. Two of the lesions (66.6%) showed arterial phase peripheral enhancement with progressive enhancement in the porto-venous and delayed phases (Figure 13). One lesion (33.3%) was ill defined, with mild heterogenous enhancement in the arterial phase persisting in portal and mild washout in the delayed phase. The patient had associated cirrhotic liver with ascites, portal vein thrombosis, and splenomegaly. This lesion was characterized as HCC on MRI, which turned out to be poorly differentiated adenocarcinoma on HPE with IHC markers suggesting cholangiocarcinoma. All 3 lesions caused intra hepatic biliary duct dilatation. On MRI, 2 cases were correctly diagnosed as cholangiocarcinoma and the other lesion was falsely diagnosed as HCC. Therefore, MRI had a sensitivity of 66.6%. On DWI, all 3 lesions showed diffusion restriction, with 1 giving the target sign, suggestive of malignancy. Therefore, DWI had a sensitivity of 100%, while the mean ADC value was 0.92 x 10^-3^ mm^2^/s.

Hepatoblastoma

**Figure 14:**
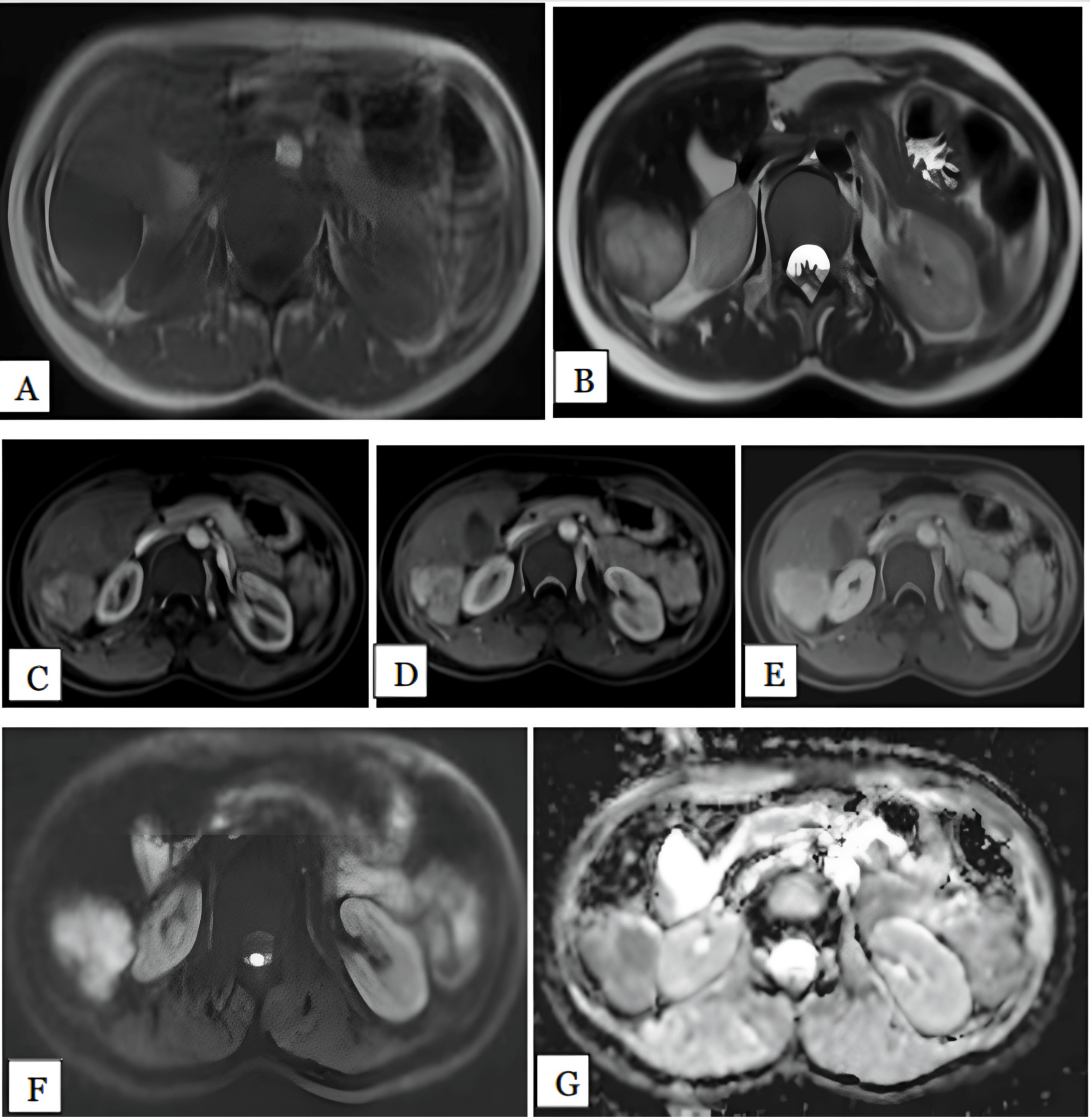
Hepatoblastoma in a 19-year-old female Axial T1 (A), T2 (B), arterial (C), portal (D) and delayed (E) phase MRI images show well-circumscribed lobulated T1 hypointense, T2 heterogenous hyperintense lesion. The lesion is showing heterogeneous enhancement on arterial and portal phases, with complete homogenous enhancement in delayed phase. HPE confirmed the diagnosis of hepatoblastoma. DWI (F) and ADC (G) maps showing the lesion is hyperintense on DWI with patchy reversal on ADC maps with mean ADC value of 1.1 x10^-3^ mm^2^/s giving target sign, suggestive of partial restriction. HPE: Histopathological examination; DWI: Diffusion-weighted imaging; ADC: Apparent diffusion coefficient

We had only one patient with hepatoblastoma: a 19-year-old female. In this case, the lesion involved segment VI of right lobe of the liver and measured 6 cm. The lesion was hypointense on T1 and hyperintense on T2-weighted sequences. The lesion showed heterogenous enhancement in the arterial phase, progression in the portal phase, and complete filling in the delayed phase (Figure 14). The lesion was characterized as a hemangioma based on imaging characteristics. Therefore, MRI had 0% sensitivity in diagnosing hepatoblastoma. However, the patient underwent MRI after receiving neoadjuvant radiotherapy and chemotherapy, which might have altered the imaging characteristics. On DWI, the lesion showed partial restriction. Therefore, DWI had a sensitivity of 50%. The mean ADC value of the lesion was 1.1 x 10^-3^ mm^2^/s.

Triphasic MRI showed a sensitivity of 95% and specificity of 100% whereas DWI showed a sensitivity of 85% and specificity of 84.76% in differentiating benign and malignant liver lesions (Table 5).

**Table 5:** Accuracy of MRI and DWI in differentiating benign and malignant liver lesions DWI: Diffusion-weighted imaging

Malignant vs benign	Triphasic MRI	DWI
Sensitivity	95	85
Specificity	100	84.76
Positive predictive value	100	79.07
Negative predictive value	96	89.28
Accuracy	97.72	84.84

## Discussion

In the present study, the simple cysts showed no diffusion restriction, with a mean ADC value of 3.1 x 10^-3^ mm^2^/s. The two simple cysts (100%) were hyperintense at lower b values (50, 400 mm^2^/s), isointense at b = 800 mm^2^/s, and showed hyperintense signal on the ADC map, which is suggestive of facilitated diffusion. Inan et al. found that at higher b values, most simple cysts (93%) are isointense with liver and show hyperintense signal on the ADC map [6]. The results of the current study are comparable with those of Inan et al. [6].

Only one case of hydatid cyst showing facilitated diffusion, with a mean ADC value of 2.6 x 10^-3^ mm^2^/s, was included. This mean ADC value was lower than the mean ADC value of the simple hepatic cyst (3.1 x 10^-3^ mm^2^/s) but higher than the mean ADC value of hemangiomas (1.74 x 10^-3^ mm^2^/s).

In this study, 70.5% of the hemangiomas showed hyperintense signal on DWI and ADC maps. The persistence of the hyperintense signal on b value of 800 mm^2^/s could be due to T2 shine through effect (validated by signal intensity on the ADC map). According to Mazroa et al., all hemangiomas show persistent hyperintense signal at increasing b values and, additionally, hyperintense or mixed signal on ADC maps, suggesting T2 shine through [7]. The mean ADC value of hemangiomas in this study was similar to the studies conducted by Namimoto et al. and Sandrasegaran et al., in which the mean ADC values of hemangiomas were 1.9 x 10^-3^ and 1.56 x 10^-3^ mm^2^/s, respectively [8,9].

The two hepatic adenomas (100%) in this study showed partial restriction, suggesting high cellular nature. They showed low ADC values, which was also the lowest of the benign liver lesions, with a mean average ADC of 1.18 x 10^-3^ mm^2^/s. This value was similar to the study by Sandrasegaran et al., in which the mean ADC value of adenomas was 1.10 x 10^-3^ mm^2^/s, owing to their cellularity [9].

Across the total 20 malignant liver lesions in our study, 70% showed restriction on DWI, owing to the high cellularity of lesions, and 30% showed partial restriction. In this study, HCC was the most common malignant liver lesion, seen in 10 patients (35.71%). On DWI, HCC showed diffusion restriction either completely (68.75%) or partially (31.25%), with an average mean ADC value of 0.96 x 10^-3^ mm^2^/s. It was lowest among all the focal liver lesions, suggesting high cellularity of the tumor, which in turn indicates the malignant nature of the lesion. This finding was similar to the study conducted by Madhu et al., in which HCC had the lowest ADC value (0.975 x 10^-3^ mm^2^/s) [10]. In their study, Helmy et al. proved that HCC had the lowest mean ADC value (1.07 x 10^-3^ mm^2^/s) of all the malignant focal liver lesions [11].

The three cholangiocarcinomas (100%) in this study showed restricted diffusion on DWI with reversal on ADC, with a mean ADC value of 0.92 x 10^-3^ mm^2^/s. This value was slightly lower than that of HCC (0.96 x 10^-3^ mm^2^/s). This result was comparable to the study conducted by Onur et al., in which the mean ADC value of cholangiocarcinoma was 1.01 x 10^-3^ mm^2^/s, while that of HCC was 1.12 x 10^-3^ mm^2^/s [12]. These diffusion properties could be attributed to accompanying fibrotic changes in the histologic structure of cholangiocarcinomas and/or relatively increased perfusion of HCCs compared to hypovascular cholangiocarcinomas. However, there was no statistically significant difference in the mean ADC values of either, which could be due to the lower sample size of cholangiocarcinomas compared to HCC.

The current study has demonstrated that the ADC map is reliable in distinguishing benign from malignant lesions. Based on the obtained average ADC value of benign hepatic lesions, 1.83 (1.23-2.43) x 10^-3^ mm^2^/s, and malignant liver lesions, 0.96 (0.80-1.12) x 10^-3^ mm^2^/s, testing showed a statistically significant difference (p < 0.01). The obtained cutoff ADC value between benign and malignant lesions was 1.301 x 10^-3^ mm^2^/s, where DWI accuracy in the overall differentiation of liver lesions was 88.63%. The results are in agreement with the study conducted by Jahic et al., who reported the best ADC cutoff value was 1.341 x 10^-3^ mm^2^/s, while the mean ADC of benign lesions was 1.88 x 10^-3^ mm^2^/s and that of malignant lesions was 1.15 x 10^-3^ mm^2^/s [13]. The average mean ADC cutoff of the compared studies was similar to the present study (Table 6). However, in the literature, the cutoff ADC values differ from study to study. This non-uniformity in the mean ADC values may be due to the differences in acquisition techniques and b values from study to study and usage of different field strength magnets

**Table 6:** Comparison of ADC values of benign and malignant lesions between the present study and other studies ADC: Apparent diffusion coefficient

Studies	Mean ADC cutoff value
Present study	1.30 × 10^-3^ mm^2^/s
Onur et al. [12]	1.23 × 10^-3^ mm^2^/s
Jahic et al. [13]	1.341 × 10^-3^ mm^2^/s

In this study, we also demonstrated the role of DWI in the differentiation of various focal liver lesions within the benign and malignant groups. Simple hepatic cysts (3.1 x 10^-3^ mm^2^/s) demonstrated higher ADC values than hydatid cysts (2.6 x 10^-3^ mm^2^/s). However, significance could not be proved because of a single case of hydatid cyst. Yalcinoz et al. demonstrated a statistically significant difference in the mean ADC values of simple cysts and hydatid cysts [14]. They attributed this difference to the denser contents of the latter, which consists of scolex, proteins, glucose, and polysaccharides.

The results of our study indicate statistically significant difference (p value <0.05) in the mean ADC values of hemangiomas (1.74 x 10^-3^ mm^2^/s) and hepatic adenomas (1.18 x 10^-3^ mm^2^/s), owing to the high cellularity of adenomas. Additionally, no significant difference was demonstrated between adenomas and malignant lesions. This observation was similar to the study by Sandrasegaran et al., in which there was no significant difference in the mean ADCs of adenomas with malignant lesions [9].

It was demonstrated that DWI had a minimal role in the differentiation of HCC from cholangiocarcinomas, as there was no significant difference (p value 91 >0.05) in the mean ADC values (0.96 and 0.92 x 10^-3^ mm^2^/s, respectively). This was in accordance with a study conducted by Onur et al., in which the mean ADC value of cholangiocarcinoma was 1.01 x 10^-3^ mm^2^/s, while that of HCC was 1.12 x 10^-3^ mm^2^/s, with no statistically significant difference [12]. Hence, triphasic MRI should be considered in addition to DWI in the differentiation of cholangiocarcinomas from HCC.

The present study also showed a statistically significant difference (p value < 0.01) in the mean ADC values of hemangiomas (1.74 +/- 0.42 x 10^-3^ mm^2^/s) and simple hepatic cysts (3.1 +/- 0.14 x 10^-3^ mm^2^/s). This observation was in accordance with a study by Tokgoz et al., where the mean ADC values of cysts were higher than hemangiomas [15].

Limitations

We had a small number of cases of hepatic adenomas, cholangiocarcinoma, and hepatoblastomas. However, these lesions were not seen as frequently as hemangioma or HCC. Histopathological confirmations of some benign hepatic lesions (simple hepatic cysts, hematomas, and few hemangiomas) were not performed. However, these lesions showed specific diagnostic imaging findings in triphasic MRI and ultrasonography, which were unchanged in follow-up imaging.

## Conclusions

DWI is a non-contrast, time-efficient, and cost-effective functional MRI technique. It is especially valuable for differentiating between benign and malignant focal liver lesions, particularly in patients with renal failure and those who are uncooperative. As DWI is a radiation-free technique, it should be considered in suspicious cases of atypical hemangiomas in young reproductive age group population to reduce the unnecessary radiation exposure. However, we could not differentiate among various malignant lesions and solid benign lesions with DWI. Therefore, in these cases, triphasic MRI should be included in addition to the DWI.
